# Genome-Scale Identification, Phylogeny, Expression Profiling, and Functional Analysis of Sugarcane DUF4228 Family Involved in Drought Stress

**DOI:** 10.3390/plants15111641

**Published:** 2026-05-27

**Authors:** Ming Lian, Farhan Goher, Zhengwang Bi, Wei Zhang, Zhuqing Wang, Yinjie Cheng, Rubab Shabbir, Hailong Chang, Shengren Sun, Qinnan Wang

**Affiliations:** 1 Institute of Nanfan & Seed Industry, Guangdong Academy of Sciences, Guangzhou 510000, China; m13224607774@163.com (M.L.); goherfarhan@nwafu.edu.cn (F.G.); sdwlbzw@163.com (Z.B.); zhangwei_870904@163.com (W.Z.); zhuqingemail@163.com (Z.W.); chengyinjieys@163.com (Y.C.); rubabshabbir28@gmail.com (R.S.); hl2004@126.com (H.C.); 2 School of Tropical Agriculture and Forestry, Hainan University, Haikou 571101, China; 3 National Key Laboratory for Tropical Crop Breeding, Sanya 572024, China; 4 National Engineering Research Center for Sugarcane, Fujian Agriculture and Forestry University, Fuzhou 350002, China

**Keywords:** sugarcane, *ScDUF4228* gene family, abiotic stress, expression pattern, yeast assay

## Abstract

Domain of unknown function (DUF) proteins play important roles in plant responses to biotic and abiotic stresses. DUF4228 proteins, as members of the DUF superfamily, are widely present in plants and exert significant functions under various stress conditions. Sugarcane is an important economic and energy crop in China. However, the role of DUF4228 family members in sugarcane has not been reported. In this study, 126 *ScDUF4228* genes were identified for the first time in the genome of the modern cultivar XTT22 and systematically named based on their chromosomal locations (*ScDUF4228-1* to *ScDUF4228-126*). These genes are located on 7 chromosomes of the XTT22 genome, exhibiting an overall uneven distribution. Phylogenetic analysis revealed that the 126 ScDUF4228 proteins can be divided into 9 groups (I–IX). Gene structure analysis indicated that most ScDUF4228 family members lack introns or contain only 1–2 introns, and all members possess a complete DUF4228 domain. Ka/Ks analysis demonstrated that the family as a whole has undergone purifying selection (Ka/Ks < 1), indicating high functional conservation during evolution. Cross-species collinearity analysis showed significant species-specific expansion of the *DUF4228* gene family in the Poaceae (particularly in sugarcane and its close relatives), a phenomenon not prominently observed in dicotyledons. Analysis of tissue expression patterns, developmental stages, and diurnal rhythms revealed that the spatiotemporal expression profiles of the 126 *ScDUF4228* family members vary, suggesting they may function individually or synergistically during different developmental periods in sugarcane. Yeast medium assay depicted that three members of the ScDUF4228 (*ScDUF4228-7*, *ScDUF4228-18*, *ScDUF4228-23*) family had significant resistance potential under drought stresses. Furthermore, transcriptome analysis after drought treatment showed that ScDUF4228-23 exhibited the most significant upregulation, suggesting it may be a key gene in sugarcane’s response to drought. These results suggest that the *DUF4228* gene family has undergone dramatic expansion in sugarcane and may play a crucial regulatory role in drought stress responses. This study provides the necessary molecular foundation for further exploring the functions of ScDUF4228 family members.

## 1. Introduction

Domain of unknown function (DUF) proteins contain at least one highly conserved DUF domain and are widely distributed in plants [1]. Currently, the Pfam database contains more than 4000 DUF proteins and uncharacterized protein families (UPF), accounting for about 23% of known domains [2]. Chris Ponting first named the DUF structure field when he incorporated DUF1 and DUF2 into the Smart database, and proposed using the “DUF + number” method to name these structure fields [3]. Systematic structural analysis suggests that many DUFs may originate from extreme diversity and novel functionalization of known protein domains [4]. The human Dicer endonuclease has a protein domain called DUF283, which exhibits high sequence similarity to the double-stranded ribonucleic acid (RNA) binding domain. This domain can bind to single-stranded nucleic acids and promote base pairing between two complementary RNA or deoxyribonucleic acid (DNA) strands [5]. In addition, studies have shown that some DUF proteins contain two or more domains. For example, the DUF1470 protein contains an N-terminal ABATE domain (Alpha-Beta-hairpin-Alpha TandEm) and a C-terminal treble-clef-like zinc finger domain [6].

Members of the DUF family play a variety of physiological and biochemical regulatory roles in plants, participating in the regulation of plant cell wall development, plant growth and development, and flower and fruit development [7]. These regulatory processes have been discovered and validated in plants such as *Arabidopsis thaliana* [8], rice (*Oryza sativa*) [9], and Chinese cabbage (*Brassica rapa* ssp. chinensis) [10]. Furthermore, studies have shown that DUF family members also play an important role in resisting biotic and abiotic stresses [11,12,13]. For example, silencing of the rice DUF500 family member *OsDUF500* showed stronger resistance to rice bacterial blight [14] and plays a negative regulatory role in rice bacterial blight resistance [14]. In another study, the sensitivity of transgenic plants to high salt and PEG6000 stressors can be increased by overexpressing the rice DUF966 family gene *OsDSR2* [15]. These findings suggest the diversification of DUF protein functions, and further exploration and utilization of these functions can help improve crop adaptability to adverse environments and contribute to the sustainable development of the agricultural economy.

The DUF4228 protein is a member of the DUF superfamily, which contains a disordered region-containing domain (PADRE), a domain unique to plants. PADRE is typically found in small, single-domain proteins with a bipartite architecture, containing a conserved motif at its N-terminus and a naturally disordered sequence with multiple phosphorylation sites at its C-terminus [13]. The DUF4228 homolog is widespread in plants, and its function has been extensively characterized. There are 16 DUF4228 family members in *Arabidopsis thaliana* [16]. Their expression levels differ significantly in response to osmotic or low-temperature stress, suggesting that the *DUF4228* gene may play a certain regulatory role in *Arabidopsis thaliana*’s response to abiotic stress [16]. Under drought and salt stress, soybean (*Glycine max*) lines overexpressing *GmDUF4228-70* showed enhanced proline content, relative water content (RWC), and chlorophyll content in leaves, and reduced malondialdehyde (MDA), H_2_O_2_, and •O_2_^−^ content. Overexpression of this gene improves soybean tolerance to drought and salt [17]. The transcript of the *CiDUF4228-3* gene in *Caragana intermedia* was significantly upregulated under dehydration, low-temperature, and drought conditions, suggesting that it may be involved in related abiotic stress responses [18]. Under heat and salt stress, the expression levels of the *StDUF4228-4* and *StDUF4228-21* genes in potatoes were high. In addition, the expression of multiple *StDUF4228* genes was significantly upregulated by IAA and ABA treatments, suggesting that *StDUF4228* genes may play a synergistic role in potatoes under abiotic stress [19].

Sugarcane (*Saccharum* spp.) is an important economic crop that plays an irreplaceable role in sugar production and energy manufacturing. Its by-products are used in multiple fields, including skin care products, pharmaceuticals, agriculture, and industry [20]. Sugarcane is mainly grown in tropical and subtropical regions. At the same time, China’s main sugarcane-producing areas are mainly distributed in rainfed arid and semi-arid slopes. The shallow topsoil, limited water resources, poor irrigation conditions, and uneven rainfall distribution exacerbate drought stress, making it the primary factor limiting sugarcane yield [21,22]. Drought stress inhibits sugarcane growth through multiple pathways, hinders root development and water and nutrient absorption, disrupts plant physiological metabolic homeostasis, leading to abnormal morphology and structure such as leaf yellowing and curling, weakens photosynthesis, reduces leaf and stem growth, and ultimately results in reduced sugarcane yield [23,24]. Therefore, enhancing cultivar drought stress tolerance is crucial for increasing sugarcane yield [25].

Recently, the first fully annotated polyploid reference genomes of the sugarcane complex Saccharum rufpilum/Erianthus rufpilum and the modern cultivar XTT22 have been published, providing valuable resources for sugarcane genome bioinformatics analysis [26,27]. Members of the *DUF4228* gene family exhibit significant responses to drought, salt, and other stresses in plants such as *Arabidopsis*, soybean, and potato. They may participate in related response processes in synergy or independently during abiotic stresses. However, the function of this family in sugarcane has not yet been reported. This study used the XTT22 genome as the research object and identified 126 *ScDUF4228* genes for the first time in the XTT22 genome. A comprehensive bioinformatics analysis was conducted on their physicochemical properties, phylogenetic relationships, conserved motifs, and chromosome distribution. Furthermore, by combining the spatiotemporal expression of *ScDUF4228* genes, transcriptomic changes during drought and diurnal rhythms, and qRT-PCR, the potential functions of *ScDUF4228* genes in sugarcane drought resistance were comprehensively analyzed. This research lays a foundation for elucidating the biological functions of the *DUF4228* gene family in polyploid sugarcane. It provides valuable candidate gene resources for subsequent drought-resistant molecular breeding.

## 2. Results

### 2.1. Identification of Members of the Sugarcane ScDUF4228 Gene Family and Analysis of Their Evolutionary Relationship

Using protein sequences of DUF4228 from *Arabidopsis thaliana*, maize (*Zea mays*), sorghum (*Sorghum bicolour*), soybean (*Glycine max*), and *Erianthus rufipilus* as references, this study identified 126 *DUF4228* genes in XTT22 through comparative screening. All of these gene sequences contain the PADRE conserved domain. Based on their chromosomal distribution, they were named *ScDUF4228-1* to *ScDUF4228-126*. The amino acid (AA) sequence lengths of the *ScDUF4228* genes ranged from 94 to 360 AAs, the relative molecular masses of 10.02 to 39.32 kDa, with the predicted isoelectric point ranging from 5.04 to 11.51 (Table S1).

**Figure 1:**
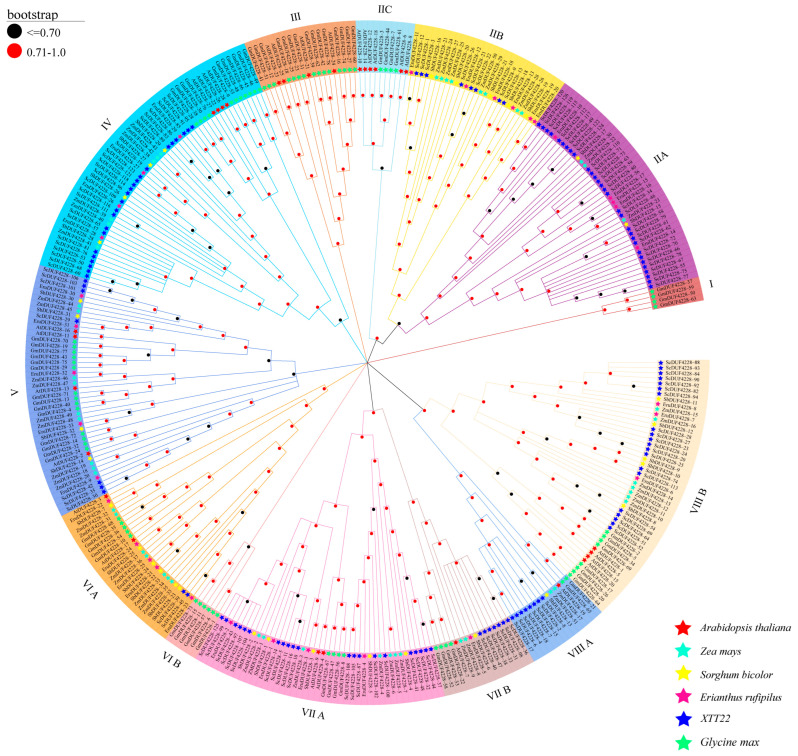
The phylogenetic relationship of DUF4228 was constructed using the maximum likelihood (ML) method for *Arabidopsis thaliana*, maize, sorghum, soybean, *Erianthus rufipilus*, and XTT22, dividing them into 8 groups (I–VIII). Node support was evaluated using a 1000-replicate bootstrap. The tree was further drawn with Evolview. A different colour represents each subgroup, and a star of a different colour represents each species.

The amino acid sequences of 349 members of the DUF4228 family, including *Arabidopsis thaliana* (AtDUF4228, 28 gene members), maize (ZmDUF4228, 53 gene members), sorghum (SbDUF4228, 33 gene members), *Erianthus rufipilus* (EruDUF4228, 33 gene members), soybean (GmDUF4228, 78 gene members), and XTT22 (ScDUF4228), were compared to assess their evolutionary relationships. Phylogenetic analysis divided these genes into eight groups (I-VIII), with group II comprising three subgroups (IIA, IIB, IIC), and groups VI, VII, and VIII each comprising two subgroups, for a total of 13 subgroups (Figure 1).

### 2.2. Chromosomal Localization and Gene Replication of the ScDUF4228 Gene Family

**Figure 2:**
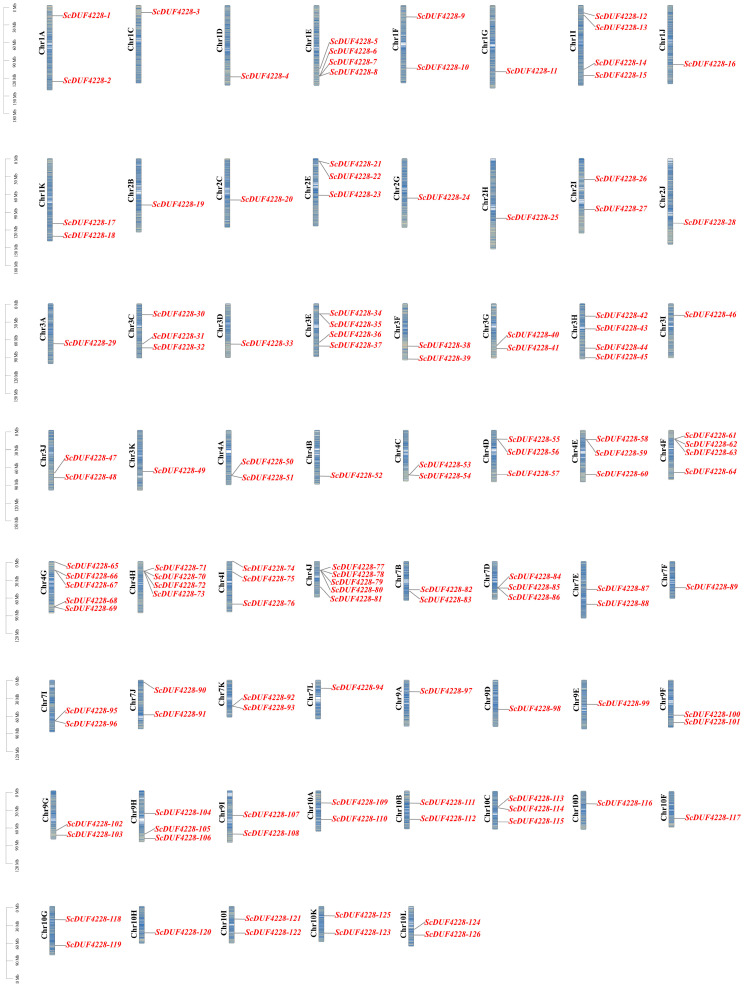
Distribution of the 126 *ScDUF4228* genes on chromosome XTT22. The scale bar indicates the length (Mb) of chromosome XTT22. Red words represent different *ScDUF4228* genes.

**Figure 3:**
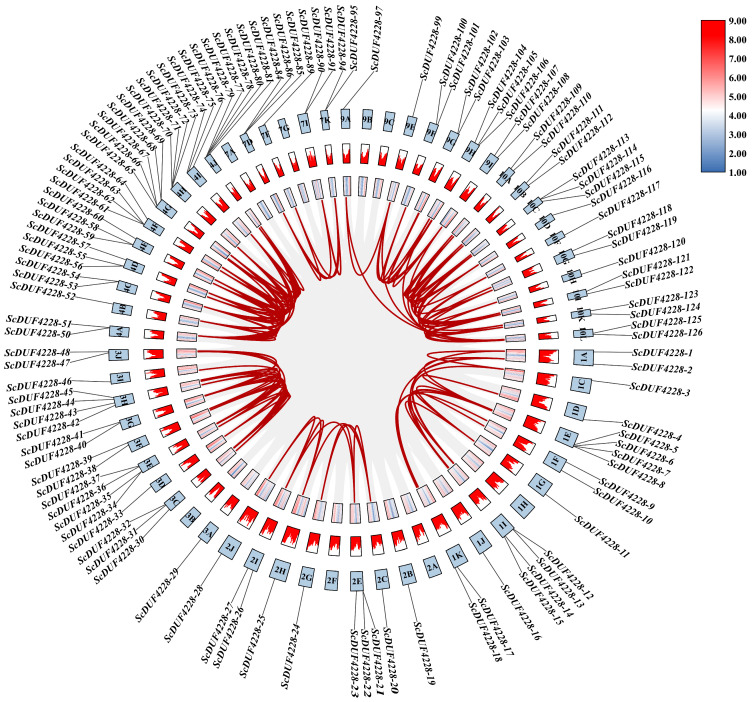
Collinearity analysis of the *ScDUF4228* gene family. Chromosomes 1, 2, 3, 4, 7, 9, and 10 are represented by blue rectangles. The red rectangle heatmap represents the gene density of the chromosomes, the grey lines represent collinear gene pairs in the XTT22 genome, and the red lines between chromosomes represent segment duplications of the *ScDUF4228* genes.

One hundred and twenty-six *ScDUF4228* genes are unevenly distributed across the seven chromosomes of XTT22, with the highest number of genes (32) on chromosome 4. Except for chromosomes Chr 4G and Chr 4J (Figure 2). A Circos visualization was constructed based on the XTT22 genome assembly, mapping 126 ScDUF4228 family genes to homologous chromosome sets and showing clear clustering on chromosomes 3, 4, and 10 (Figure 3). Parallel homology relationships inferred by MCScanX formed three high-density repeat clusters, identifying 134 gene pairs (connected by red strings), indicating that fragment duplication and whole-genome duplication (WGD) are the main drivers of expansion. The nonsynonymous (Ka)/synonymous (Ks) ratio of each gene pair was calculated using the Ka/Ks-Calculator tool (listed in Table S2) to explore the evolutionary constraints of the *ScDUF4228* gene. The results showed an average Ka/Ks of 0.64 per gene pair, with 107 pairs showing Ka/Ks < 1 and 19 pairs showing Ka/Ks > 1. This indicates that most members of the ScDUF4228 gene family have undergone purifying selection (negative selection) during evolution, with most harmful mutations being eliminated, resulting in very high functional conservation of genes.

### 2.3. Analysis of Gene Structure, Conserved Domains and Conserved Motifs of Members of the ScDUF4228 Gene Family

**Figure 4:**
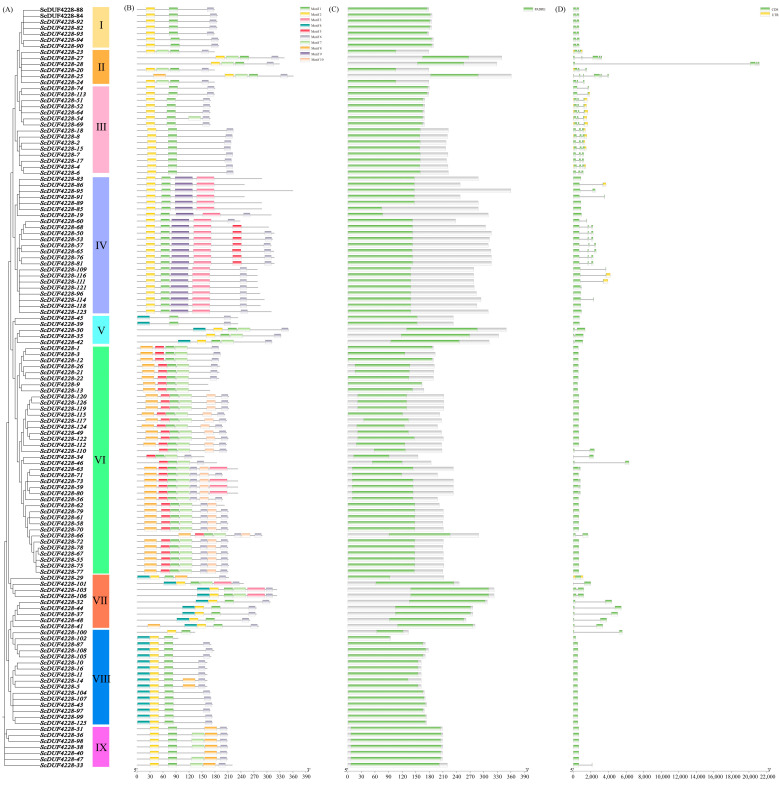
Conserved motifs, conserved domains, and gene structure predictions for 126 members of the *ScDUF4228* gene family. (**A**) Phylogenetic tree of the ScDUF4228 protein constructed using the maximum likelihood (ML) method and divided into nine subgroups. (**B**) Distribution of conserved motifs in the *ScDUF4228* gene family; colored boxes of different colors represent 10 conserved motifs. (**C**) Prediction of conserved domains of the *ScDUF4228* gene; green squares represent the PADRE conserved domain. (**D**) Exon/intron organization of the *ScDUF4228* gene family. Green squares represent exons, black lines represent introns, and yellow squares represent the UTR regions of the *ScDUF4228* gene family. The scale at the bottom is used to infer exon lengths.

A phylogenetic tree of 126 ScDUF4228 amino acid sequences in XTT22 was constructed using the maximum likelihood (ML) method, and the sequences were clustered into 9 subgroups (Figure 4A). Conserved motif prediction of the above amino acid sequences showed that the top 10 most conserved Sites per Motif ranged from 2 to 126 amino acids, with motif 1 being the most conserved and motif 9 being the least conserved. Sequence logos for the conserved motifs of ScDUF4228 proteins in plants in Figure S1. The ScDUF4228 gene, located on the same evolutionary branch, also displayed highly similar conserved motif patterns (Figure 4B). Conserved domain prediction results showed that all ScDUF4228 proteins possessed the PADRE conserved domain (Figure 4C), indicating that the ScDUF4228 protein domains are highly conserved. Structural analysis of the ScDUF4228 family gene sequences revealed that most ScDUF4228 genes contained 1-2 introns or no introns. ScDUF4228-25 contained 5 introns, 59 ScDUF4228 genes (46.8%) had no detected introns, 39 ScDUF4228 genes (30.9%) had 1 intron, and 23 genes (18.2%) had 2 introns (Figure 4D).

### 2.4. Cis-Acting Element Prediction of the ScDUF4228 Gene Family

**Figure 5:**
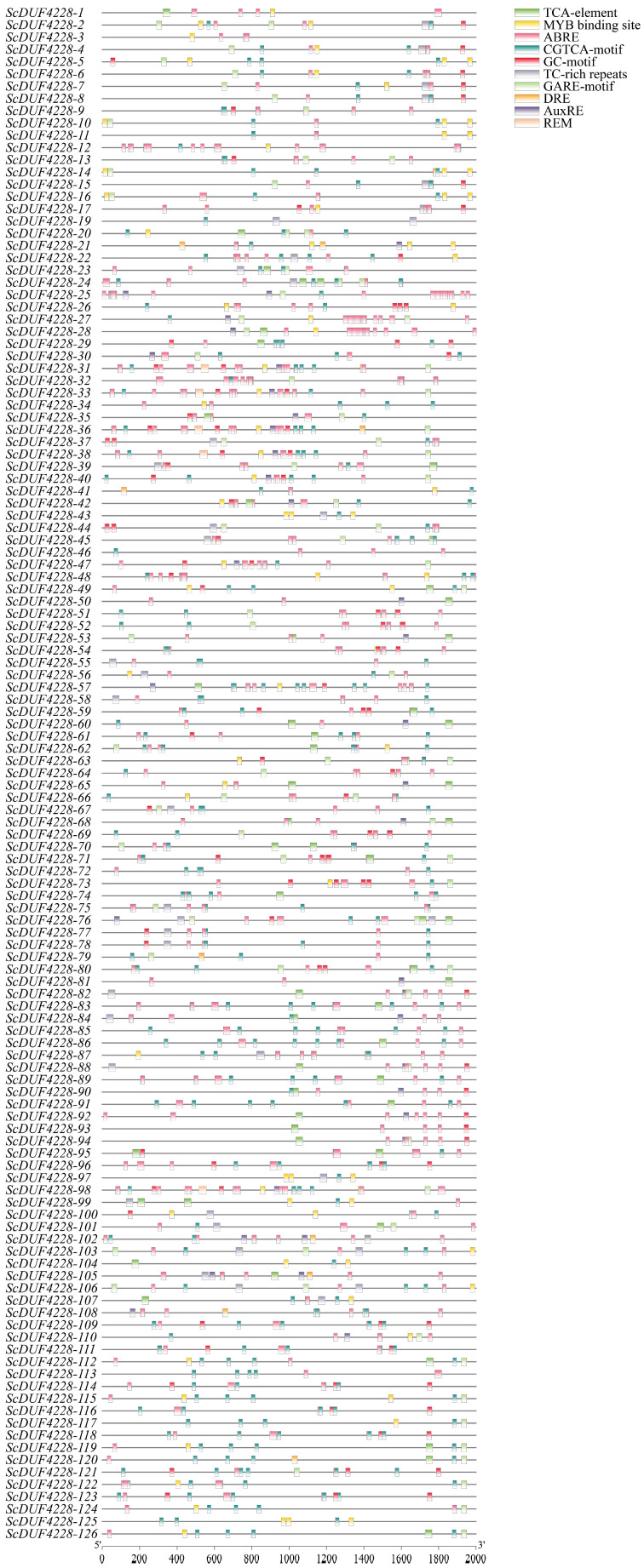
PlantCare was used to predict the distribution of *cis*-regulatory elements in the first 2 kb region of the *ScDUF4228* gene family promoter. The colored boxes on the right represent *cis*-regulatory elements, each with a specific function.

To explore the possible biological functions of members of the *ScDUF4228* gene family, this study used the PlantCARE database to analyze the *cis*-acting elements within the first 2000 bp upstream of each gene start site. Ten specific *cis*-elements were identified in the promoter region, mainly those related to growth and development, hormone response, and abiotic stress response (Figure 5). The abiotic stress elements mainly include drought response elements (MYB binding site) and defence and stress response elements (TC-rich repeats); hormone response elements include auxin (AuxRE), methyl jasmonate (CGTCA-motif), gibberellin (GARE-motif), abscisic acid (ABRE), and salicylic acid (TCA-element) response elements. The diversity of *cis*-acting elements suggests that the *ScDUF4228* gene family is widely involved in diverse biological processes and may play an important role, especially in drought stress and hormone response pathways.

### 2.5. Interspecific Collinearity Analysis of the Sugarcane DUF4228 Gene Family

**Figure 6:**
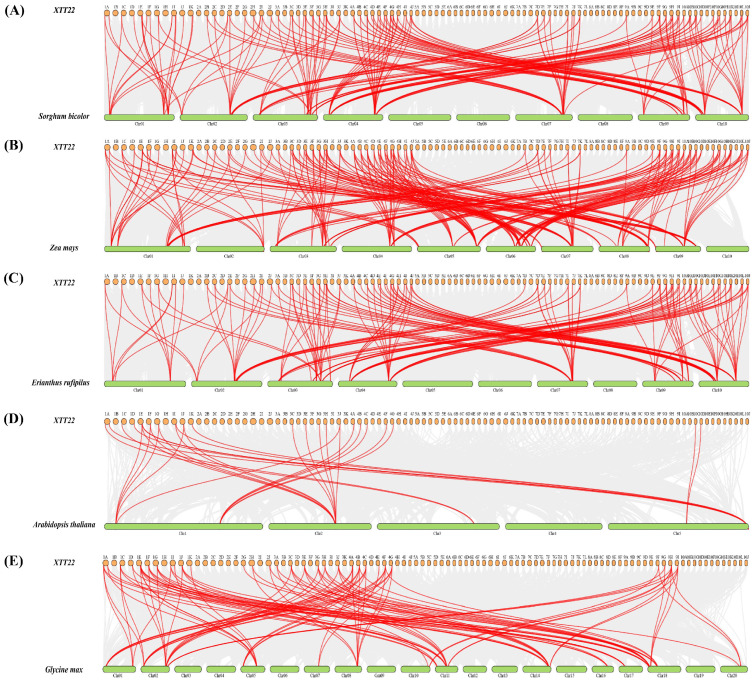
Orthologous homology analysis of the XTT22 genome with the *ScDUF4228* gene family from five other plant species. Gray lines represent genes homologous to the XTT22 genome from other genomes, while red lines depict orthologous pairs of the *ScDUF4228* gene. Orthologous homology analysis of the XTT22 genome with the *ScDUF4228* genes from the genomes of *Sorghum bicolor* (**A**), *Zea mays* (**B**), *Erianthus rufipilus* (**C**), *Arabidopsis thaliana* (**D**), and *Glycine max* (**E**).

To further trace the evolutionary origin of the sugarcane *DUF4228* gene family, whole-genome collinearity analysis was performed with XTT22 as the reference genome and five representative species (Figure 6). The results showed that this genome had 177, 227 and 156 homologous gene pairs (red lines) with the genomes of sorghum, maize and the closely related wild species *Erianthus rufipilus* forming dense and orderly collinear blocks, reflecting the whole-genome duplication shared by the Poaceae family and the significant expansion caused by subsequent polyploidization of the “Saccharum” genus (Figure 6A–C). In stark contrast, the XTT22 genome shares 30 and 100 homologous gene pairs with dicotyledonous plants *Arabidopsis thaliana* and soybean, respectively, indicating a relatively small number of homologous gene pairs (Figure 6D,E). These results suggest that the *ScDUF4228* gene family has undergone significant specific amplification compared to grasses, especially sugarcane and its close relatives, which is highly consistent with the dramatic increase in copy number in the sugarcane genome.

### 2.6. Expression Profiles and Circadian Rhythms of ScDUF4228 Family Genes in Various Tissues of XTT22

**Figure 7:**
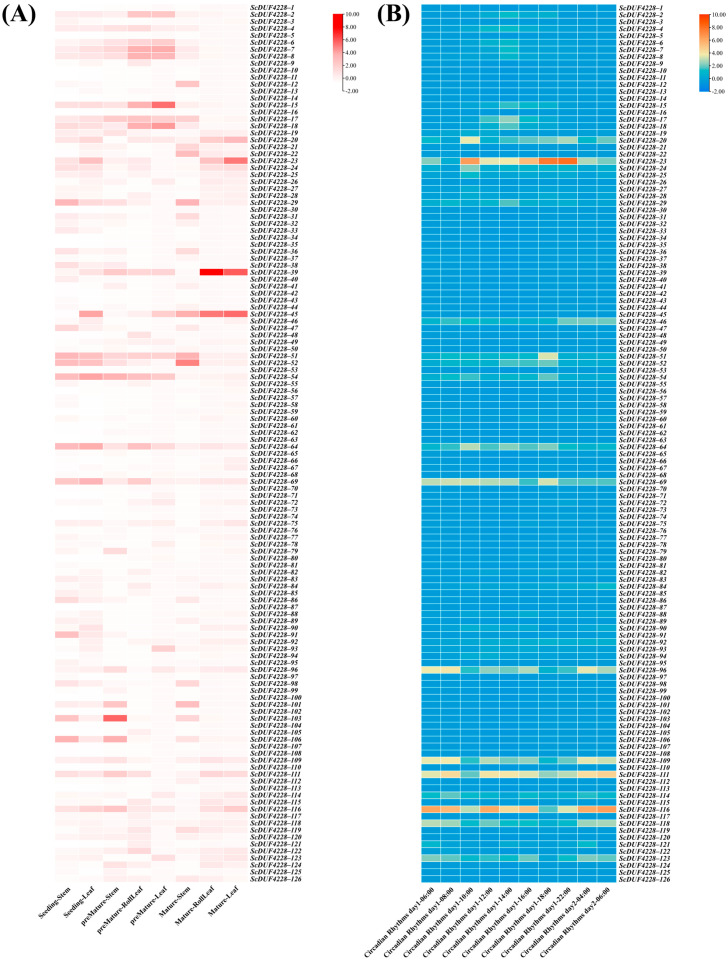
Heatmaps of *ScDUF4228* family gene expression levels in various tissues and their diurnal rhythms. (**A**) Heatmaps of *ScDUF4228* family gene expression in different tissues and organs (Mature-Leaf: mature (12 months) leaf, Mature-Roll Leaf: mature (12 months) rolled leaf, Mature-stem: mature (12 months) stem node, preMature-Leaf: immature (9 months) leaf, preMature-Roll Leaf: Immature (9 months) curled leaf, preMature-stem: Immature (9 months) stem node, Seeding-leaf: Seedling stage (35 days) leaf, Seeding-stem: Seedling stage (35 days) stem, red and white represent high and low expression levels, respectively. (**B**) Circadian rhythm expression heatmap of *ScDUF4228* family genes (mature SES208, samples of the first leaf were collected every 2 h from 6:00 to 18:00. Subsequently, samples were collected at 20:00 and 22:00 on the first day and at 6:00 on the second day.). Scale bar: blue transitioning to red indicates expression levels from low to high.

To investigate the expression patterns of the *ScDUF4228* family of genes in different tis sues during development, RNA-seq data of this family of genes in eight sugarcane tissues and organs (including leaves and stems of SES208 in the seedling stage (35 days old); rolled leaves, leaves, and stem nodes in the immature stage (9 months old); and rolled leaves, leaves, and stem nodes in the mature stage (12 months old)) were extracted from publicly available RNA-seq data in the Saccharum genome database. The expression profiles of the *ScDUF4228* gene family in different tissues were analyzed (Figure 7A). The results showed that *ScDUF4228-20*, *ScDUF4228*-*23*, *ScDUF4228*-*39*, and *ScDUF4228*-*45* genes were highly expressed in mature and rolled leaves, whereas *ScDUF4228-1*, *ScDUF4228*-*7*, *ScDUF4228*-*8*, *ScDUF4228*-*15*, and *ScDUF4228*-*17* were highly expressed only in immature and rolled leaves. *ScDUF4228-51*, *ScDUF4228*-*52*, *ScDUF4228*-*64*, and *ScDUF4228*-*69* genes were highly expressed in seedling leaves and stems (based on the criterion of “expression fold change ≥ 3”).

Circadian rhythm expression analysis of *ScDUF4228* family genes was performed using RNA-seq data obtained from the Saccharum genome database (Figure 7B). Genes *ScDUF4228-23*, *ScDUF4228*-*96*, *ScDUF4228*-*109*, *ScDUF4228*-*111*, *ScDUF4228*-*116*, *ScDUF4228*-*118*, and *ScDUF4228*-*123* showed high expression levels throughout the day, with the highest expression levels occurring mainly during the midnight-early morning period (22:00 on the first night to 4:00 on the second morning). *ScDUF4228-69* was upregulated only between 6:00 and 8:00, whereas *ScDUF4228-2*, *ScDUF4228*-*4*, *ScDUF4228*-*6*, *ScDUF4228*-*7*, *ScDUF4228*-*8*, *ScDUF4228*-*15*, *ScDUF4228*-*17*, and *ScDUF4228*-*18* showed upregulation during the midday-afternoon period (12:00-16:00), with lower expression at other time points. These results indicate that different members of the ScDUF4228 family may participate individually or synergistically in distinct growth and developmental processes in sugarcane plants.

### 2.7. Expression Pattern Analysis of the ScDUF4228 Gene Family Under Drought Stress

**Figure 8:**
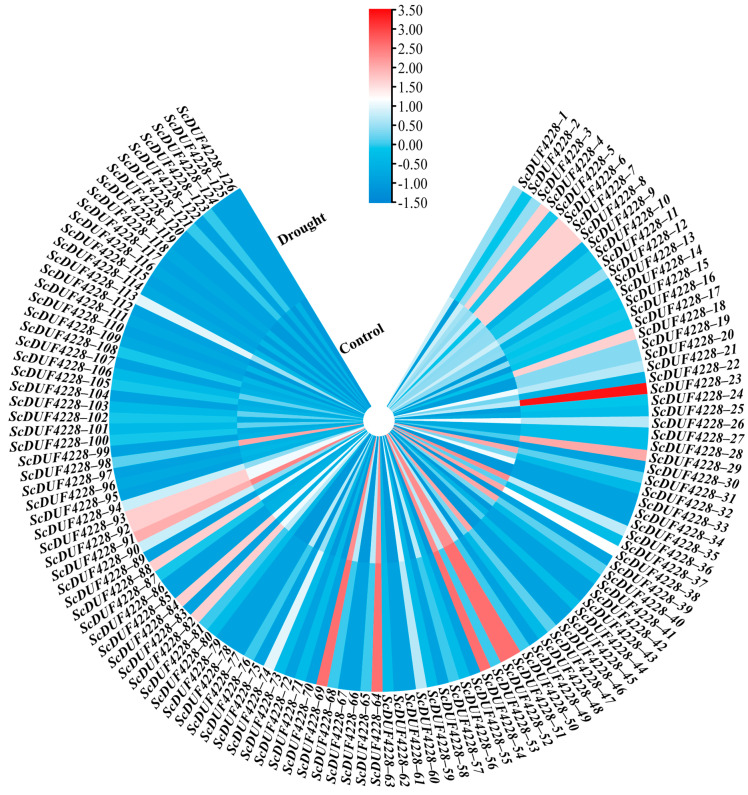
Heatmap of differential expression of the *ScDUF4228* genes under drought stress. The scale, comprising blue and red colors, transitions indicate increasing expression levels from blue to red. The log2-transformed FPKM value was used to represent the gene expression levels, and the expression was represented with the heatmap tool in TBtools-II (version 2.481) software.

**Figure 9:**
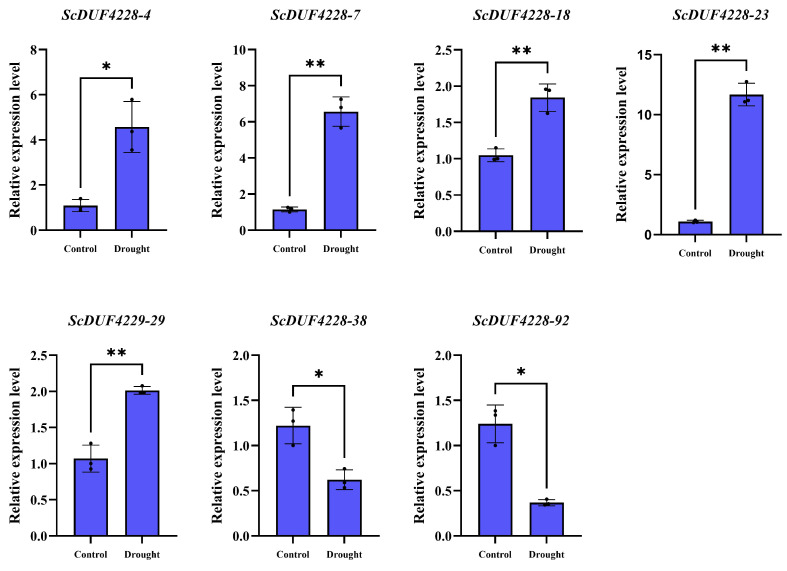
The relative expression level of the *ScDUF4228* genes in XTT22 under drought stress conditions. * indicates a significant difference (*p* < 0.05); ** indicates a highly significant difference (*p* < 0.01).

To investigate the expression patterns of the *ScDUF4228* gene family under drought stress, RNA-Seq transcriptome data were used to analyze its expression. As shown in Figure 8, most *ScDUF4228* genes were induced to express under drought stress. A total of 23 genes were upregulated under drought stress (based on the fold change ≥ 2), among which *ScDUF4228-4*, *ScDUF4228*-*6*, *ScDUF4228*-*7*, *ScDUF4228*-*18*, *ScDUF4228*-*23*, and *ScDUF4228*-*29* showed the highest upregulated expression levels, with fold changes of 3.3 (*ScDUF4228-4*) and −4.1 (*ScDUF4228-23*) times compared to the control (Figure 8). Among them, *ScDUF4228-23* showed the highest fold change in expression. The expression of 75 genes was downregulated (based on the criterion of “change in expression ≤ 1”), including *ScDUF4228-31*, *ScDUF4228*-*35*, *ScDUF4228*-*38*, *ScDUF4228*-*40*, *ScDUF4228*-*92*, and *ScDUF4228*-*113*, with expression levels downregulated by 1.31 (*ScDUF4228-113*) to 20 (*ScDUF4228-31*) times compared to the control (Figure 8). This may indicate their potential negative resistance role during drought stress. Those highly downregulated and upregulated *ScDUF4228* family genes are further validated through RT-qPCR assay. So, the expression patterns of *ScDUF4228* genes, *ScDUF4228-4*, *ScDUF4228-7*, *ScDUF4228-18*, *ScDUF4228-23*, *ScDUF4228-29*, *ScDUF4228-38,* and *ScDUF4228-92* in XTT22 leaves under drought stress were analyzed (Figure 9). The results showed that the expression levels of *ScDUF4228-4*, *ScDUF4228-7*, *ScDUF4228-18*, and *ScDUF4228-23* were all significantly upregulated under drought treatment compared with the control (Figure 9). Among all, *ScDUF4228-23* showed the highest upregulation (8.69-fold increase relative to the control), followed by *ScDUF4228-7* (6.56-fold), *ScDUF4228-4* (4.57-fold), and *ScDUF4228-18* (1.84-fold). The highest induction of *ScDUF4228-23* suggested its resistance role during drought stress, which could serve as a potential candidate in resistance breeding. *ScDUF4228-29* initially increased by approximately 2.01-fold after drought treatment and then decreased, which may indicate its transient resistance role under drought treatment (Figure 9). The expression levels of *ScDUF4228-38* and *ScDUF4228-92* decreased after drought treatment by 1.6-fold and 2.72-fold, respectively. In summary, the expression levels of the *ScDUF4228* gene family varied significantly across different time points after drought treatment. Transcriptome analysis revealed that members of the *ScDUF4228* gene family in XTT22 exhibited a broad response to drought stress; however, the specific functions and response mechanisms of individual genes require further investigation.

### 2.8. Functional Analysis of ScDUF4228 Genes in Yeast Media

**Figure 10:**
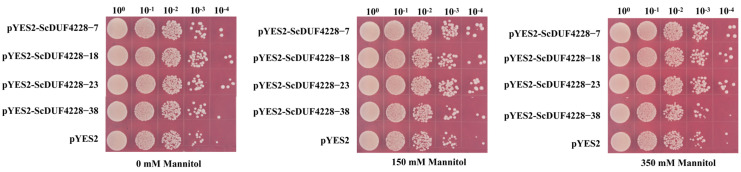
The expression of *ScDUF4228* genes in yeast transformants (INVSc1) enhances tolerance to abiotic stresses.

To analyze the functional characteristics of ScDUF4228, four genes were selected on the basis of their expression under drought conditions in the RT-qPCR assay. *ScDUF4228-7*, *ScDUF4228-18*, and *ScDUF4228-23* have shown significant upregulation compared to the control. While *ScDUF4228-38* was selected as it showed significant downregulation compared to the control. Therefore, a yeast assay transfected by the plasmid carrying *ScDUF4228-7*, *ScDUF4228-18*, *ScDUF4228-23*, and *ScDUF4228-38* genes was conducted under mannitol 0 mM, 150 mM, and 350 mM stresses. No significant difference in yeast cell growth was detected between transfections with the *ScDUF4228-38* plasmid and empty vector on SG medium or under both of the mannitol (150 mM, 350 mM) stresses (Figure 10). On the other hand, there was a significant difference in the growth of yeast with plasmids carrying *ScDUF4228-7*, *ScDUF4228-18*, and *ScDUF4228-23* genes under mannitol stress compared to the control (Figure 10). Hence, it was suggested that *ScDUF4228-7*, *ScDUF4228-18*, and *ScDUF4228-23* genes may confer improved tolerance against the mannitol stressors.

## 3. Discussion

Unknown functional domains (DUFs) are widely distributed in plant genomes, and increasing evidence suggests that they play important roles in plant development, reproduction, and resistance to abiotic stresses. These proteins can act as regulatory factors, influencing plant stress resistance by affecting IAA and ABA synthesis, calmodulin expression, chlorophyll content, and polysaccharide metabolism [28]. The DUF4228 family, a member of the DUF superfamily, has been shown to play important roles in abiotic stress responses in plants such as Arabidopsis, soybean, and Gossypium. Overexpression of the *AtPADRE13* reduces salt stress tolerance in *Arabidopsis* [29], overexpression of the *GmDUF4228-70* in soybean increases its tolerance to drought and salt stress [17], and silencing the *GhDUF4228-67* in gossypium reduces its salt tolerance [30]. This study used protein sequences of DUF4228 from *Arabidopsis thaliana*, maize (*Zea mays*), sorghum (*Sorghum bicolour*), soybean (*Glycine max*), and *Erianthus rufipilus* as references, and identified 126 *ScDUF4228* genes for the first time in the genome of the modern hybrid XTT22 (Figure 1). These genes are unevenly distributed across the seven chromosomes of XTT22. Analysis of conserved motifs, conserved domains, and gene structure of the *ScDUF4228* family members revealed that the 126 members can be clustered into nine subgroups (Figure 4). The gene structures of the different subgroups of *ScDUF4228* genes differed significantly, yet each subgroup exhibited high conservation. Most members lacked introns or contained only 1-2 introns, similar to the results of gene analysis of the DUF4228 family in Gossypium species [30]. Conserved domain analysis revealed that all ScDUF4228 proteins contained the complete DUF4228 core domain (PF14009), consistent with Yasir Sharif’s findings [19] (Figure 4). The specific amplification levels of the 126 *ScDUF4228* genes in XTT22 were significantly higher than those in *Arabidopsis* [16], potato [19], and soybean [17], which may be related to the complex polyploid background of sugarcane. This amplification mainly stemmed from genome-wide duplication and fragment duplication events, rather than tandem duplication. During intraspecific gene amplification in XTT22, the Ka/Ks ratio of most *ScDUF4228* tandem gene pairs was less than 1, indicating purifying selection and maintenance of core functional conservation (Table S2).

*Cis*-acting elements are among the major regulators of gene expression, regulating the expression of related genes in response to growth and development processes and environmental changes [31]. The promoter region of the *ScDUF4228* genes contains several abiotic stress-related *cis*-acting elements, including a drought-induced MYB binding site [32,33] and hormone-responsive *cis*-acting elements [34], The abiotic stress elements mainly include drought response elements (MYB binding site) and defence and stress response elements (TC-rich repeats); hormone response elements include auxin (AuxRE), methyl jasmonate (CGTCA-motif), gibberellin (GARE-motif), abscisic acid (ABRE), and salicylic acid (TCA-element) response elements. (Figure 5), which are similar to promoter elements in rice (*OsDUF506*) [35], alfalfa (*MsDUF1644*) [36], and tobacco (NtDUF668) [37]. High number of enriched abiotic stress responsive *cis* elements in sugarcane DUF4228 family suggested it is crucial role in abiotic stress resistance [38,39].

Expression pattern analysis indicated that members of the *ScDUF4228* gene family may regulate diurnal rhythm changes throughout the sugarcane growth cycle. RNA-seq data obtained from the Saccharum genome database suggest that several members of the ScDUF4228 family may engage in different growth and developmental processes in sugarcane plants either independently or in combination (Figure 7B). RNA-seq data revealed that under drought stress, many *ScDUF4228* genes expression were elevated. Of these, *ScDUF4228-4*, *ScDUF4228-6*, *ScDUF4228-7*, *ScDUF4228-18*, *ScDUF4228-23*, and *ScDUF4228-29* had the highest levels of increased expression when compared to the control (Figure 8). Furthermore, the findings demonstrated that under drought treatment, the expression levels of *ScDUF4228-4*, *ScDUF4228-7*, *ScDUF4228-18*, and *ScDUF4228-23* were considerably increased in comparison to the control upon validation with RT-qPCR analysis (Figure 9). Notably, the expression levels of several *ScDUF4228* genes changed significantly under drought stress, with *ScDUF4228-23* increasing 4.1-fold compared to the control. RT-qPCR results further confirmed that *ScDUF4228-23* significantly responds to drought stress, suggesting its potential role in the sugarcane-drought interaction. Furthermore, in the yeast assay, *ScDUF4228-7*, *ScDUF4228-18*, and *ScDUF4228-23* showed growth patterns under drought stresses (Figure 10). These hypotheses require further validation through the development of transgenic sugarcane plants, but the aforementioned bioinformatics analysis, accompanied by expression profile and functional characterization under yeast medium, provides a reliable framework for understanding the structure and evolutionary characteristics of the sugarcane *DUF4228* gene family under drought stress.

## 4. Materials and Methods

### 4.1. Identification of Sugarcane DUF4228 Gene Family Members and Analysis of Their Physicochemical Properties

In this study, Arabidopsis genome data were obtained from the TAIR database (https://www.arabidopsis.org, accessed on 8 September 2025) [40], maize and sorghum genome data were obtained from the NCBI database (https://www.ncbi.nlm.nih.gov, accessed on 8 September 2025), soybean genome data were obtained from the SoyBase database (http://www.soybase.org, accessed on 8 September 2025) [41], and sugarcane and XTT22 genome data were obtained from the Sugarcane Genome Database (https://sugarcane.gxu.edu.cn/scdb/, accessed on 8 September 2025) [42]. To identify the ScDUF4228 protein sequence, an HMM model of the DUF4228 domain (PF14009) was downloaded from the Pfam database (http://pfam.xfam.org, accessed on 12 September 2025). Using the Arabidopsis DUF4228 protein sequence as the query sequence, HMMER 3.0 software (http://hmmer.org, accessed on 10 September 2025) was used to perform a genome-wide search on several other plants based on the DUF4228 domain. With an E-value < 1 × 10^−5^, sequences with less than 40% similarity were removed, resulting in candidate sequences *ZmDUF4228*, *SbDUF4228*, *GmDUF4228*, *EruDUF4228*, and *ScDUF4228* [43]. All candidate proteins underwent triple validation using Pfam, SMART, and NCBI CD-Search to ensure the DUF4228 domain was complete and had ≥90% coverage. The physicochemical properties of proteins were predicted using the Protein Parameter Calculator tool in TBtools-II.

### 4.2. Multiple Sequence Alignment and Phylogenetic Analysis

Phylogenetic trees were constructed using MEGA X software (version 10.2.6). First, all sequences were re-aligned using Clustal W (http://www.ebi.ac.uk/clustalw/, accessed on 12 September 2025) [44] with default parameters. Because gene family members across the above species are not completely homologous, gaps were removed during alignment to improve the reliability of the results. The phylogenetic tree calculations described above were based on maximum likelihood estimation, and node support was evaluated using a 1000-replicate bootstrap test [45]. The generated phylogenetic trees were further beautified using the online tool Evolview [46].

### 4.3. Chromosomal Localization and Homolinearity Analysis of the ScDUF4228 Gene Family

To elucidate the chromosomal location and evolutionary trajectory of the *ScDUF4228* gene family, this study performed chromosomal localization and homology analysis of its members. First, based on the XTT22 GFF format genome annotation file, TBtools was used to locate and visualize the *ScDUF4228* gene family on chromosomes [47]. Collinear gene pairs of *ScDUF4228* were identified using the MCScanX module in TBtools-II (version 2.481) and visualized using the “Advanced Circos” module [48]. The synonymous substitution rate (Ks), non-synonymous substitution rate (Ka), and Ka/Ks ratio of *ScDUF4228* gene pairs were calculated using the “Gene Location Visualize from GTF/GFF” and “Simple Ka/Ks Calculator (NG)” modules in TBtools-II software [49]. The “One step MCScanX” function was used to perform cross-genome collinearity analysis of the *ScDUF4228* genes in XTT22 and its orthologs in *Arabidopsis thaliana*, maize, sorghum, soybean, and sugarcane. The results were visualized using the “Multiple Synteny Plot” tool.

### 4.4. Prediction of Cis-Acting Elements of the ScDUF4228 Gene Family Promoter

The promoter sequence, located 2000 bp upstream of the transcription start site, was extracted from the XTT22 genome file using the Gtf/Gff3 Sequences Extract function in TBtools-II. The sequence was analyzed using the online database PlantCARE, and the resulting *cis*-regulatory elements were organized and further analyzed [50]. The results were visualized using the Basic Biosequence View tool in TBtools-II.

### 4.5. Analysis of ScDUF4228 Gene Family Structure, Conserved Motifs, Conserved Domains, and Gene Expression

The *ScDUF4228* gene sequences were extracted using the Gtf/Gff3 Sequences Extract function in TBtools-II software. Subsequently, the Visualize Gene Structure function was used to visualize the exon/intron arrangement of the *ScDUF4228* gene family. Conserved motifs of the protein encoded by the *ScDUF4228* gene were predicted using the online tool “Multiple Em for Motif Elicitation” (https://meme-suite.org, accessed on 13 September 2026) [51]. The conserved domains of the ScDUF4228 protein were analyzed using the NCBI Batch-CD-search [52] tool and visualized using the “Gene Structure View” module in TBtools-II.

### 4.6. Transcriptome Data Acquisition and Expression Analysis

Transcriptome data of sugarcane under simulated drought stress were downloaded from a previous study [53] (PRJNA975299). Tissue expression pattern and diurnal rhythm expression transcriptome data of XTT22 were obtained from the Sugarcane Genome Database. FastQC v 0.11, HISAT v2.1.0, and StringTie v1.3.4d were used to filter the original data, to get the clean reads, and to predict new genes. RNA-seq data analysis was performed using the XTT22 genome as a reference genome [27] (transcriptome data are listed in Tables S3–S5). FeatureCounts v1.6.2 was used to calculate the gene alignment and FPKM. DESeq2 v1.22.1 was used to analyze differential expression. The log2-transformed FPKM value was used to represent the gene expression level of the sample. Subsequently, the expression heatmap tool in TBtools-II software [47] was used to draw expression heatmaps to visualize the expression level of *ScDUF4228* genes. Genes showing significantly high expression under drought stress were selected for subsequent functional studies.

### 4.7. XTT22 Drought Treatment

XTT22 sets were planted in pots (buds facing upwards) containing a 3:1 (*v*/*v*) mixture of organic substrate and vermiculite in a greenhouse and cultured for 3 weeks before being subjected to drought stress. The plants were thoroughly watered the day before drought stress treatment, and any remaining water was drained the following day. Drought stress treatment was then initiated, and the water supply was stopped during the treatment period. Tissue samples from +1 leaves were collected 9 days after drought stress and rapidly frozen in liquid nitrogen before being transferred to a −80 °C freezer for subsequent RNA extraction and real-time quantitative PCR (RT-qPCR) analysis. Three biological replicates were configured for the experiment.

### 4.8. RNA Extraction and RT-qPCR Analysis 

Total RNA was extracted from the above samples using a standard RNA extraction kit (TransGen, Beijing, China). The quality and concentration of RNA were assessed by agarose gel electrophoresis and a Q5000 ultra-micro UV spectrophotometer (Quawell, San Jose, CA, USA). 0.5 μg of total RNA, digested with DNase I, was reverse-transcribed into cDNA using the PrimeScript™ RT reagent Kit with gDNA Eraser (TaKaRa, Dalian, China). cDNA amplification was then performed using TB Green Premix Ex Taq II (Tli RNase H Plus) (TaKaRa, Dalian, China) on a QuantStudio™ 5 real-time quantitative PCR system (Thermo Fisher, Waltham, MA, USA). The reaction program was as follows: pre-denaturation at 95 °C for 10 s; followed by 40 cycles (95 °C for 10 s, 60 °C for 30 s); after 40 cycles, melting curves were analyzed within the range of 60–95 °C. All RT-qPCR experiments were performed in triplicate. The relative transcriptional level of the *ScDUF4228* genes was calculated using the 2^−ΔΔCT^ method [54], and the glyceraldehyde-3-phosphate dehydrogenase (*GAPDH*) gene was selected as the internal control gene for RT-qPCR analysis. Primers are listed in Table S6.

### 4.9. Vector Construction and Stress Tolerance Assay in Yeast

The yeast expression vector pYES2/NT B was linearized by double digestion with the restriction endonucleases Hind III and EcoR I. The PCR products of the target genes ScDUF4228-7, ScDUF4228-18, ScDUF4228-23, and ScDUF4228-38, together with the linearized vector, were purified via gel extraction using a Gel Extraction Kit (Omega, Norcross, GA, USA) from a 1.5% agarose gel, and then stored at −20 °C for subsequent use. The target gene fragments were ligated into the linearized vector pYES2/NT B using a DNA Assembly Mix Plus seamless cloning kit (Lambertbio, Beijing, China). The pYES2-*ScDUF4228*-7, pYES2-*ScDUF4228*-18, pYES2-*ScDUF4228*-23, pYES2-*ScDUF4228*-38, and pYES2/NT B plasmids were transfected into the yeast INVSc1 and then were cultivated on SC-Ura medium for an entire night before being diluted with fresh medium. The 10 µL solution was spotted on the SG-Ura medium (SC-Ura medium with 2% galactose) plus 0 mM, 150 mM, and 350 mM mannitol, then cultured at 30 °C for 3 days, and the growth rates of the yeast were examined. The empty vector (pYES2/NT B) transfected into the yeast INVSc1 was used as the control. Three independent replications were performed. Primers are listed in Table S6.

## 5. Conclusions

In summary, this study identified 126 *ScDUF4228* genes in the genome of the modern sugarcane hybrid XTT22 for the first time and predicted their chromosomal locations, conserved motifs, conserved domains, gene structures, and collinearity. This dramatic expansion in a highly polyploid background was primarily mediated through genome doubling and fragment duplication events. This process underwent intense purifying selection. *Cis*-regulatory element prediction revealed that the promoter region is enriched in drought, hormone regulation, and defence-related *cis*-regulatory elements, suggesting that some members of the *ScDUF4228* gene family may be involved in abiotic stress responses. Transcriptome and qRT-PCR analyses showed that *ScDUF4228-23* was most strongly expressed under drought conditions, making it a promising candidate gene for drought resistance. Further research will validate its function using techniques such as gene overexpression and gene editing in sugarcane. These findings not only fill a gap in the study of the *ScDUF4228* gene family in sugarcane but also reveal the dynamic evolution of DUF4228 in polyploid crops and its potential key role in drought response, providing crucial candidate gene resources for sustainable sugarcane production.

## Data Availability

The original contributions presented in this study are included in thearticle/Supplementary Material. Further inquiries can be directed to the corresponding authors.

## Supplementary Materials

The following supporting information can be downloaded at: https://www.mdpi.com/article/10.3390/plants15111641/s1, Figure S1: Sequence logos for the conserved motifs of DUF4228 proteins; Table S1: Analysis of gene IDs and protein physicochemical properties of the *ScDUF4228* family genes; Table S2: Nonsynonymous (Ka)/synonymous (Ks) ratio of the *ScDUF4228* gene family; Table S3: The expression of *ScDUF4228* family genes under circadian rhythm was determined by the RNA-Seq data; Table S4: The tissue-specific expression of the *ScDUF4228* family genes was determined using RNA-Seq data; Table S5: The expression of *ScDUF4228* family genes under drought stress was determined using RNA-Seq data; Table S6: Primers for *ScDUF228* family genes under drought stress.
